# Clinical characteristics, predictors, and performance of case definition—Interim results from the WHO global respiratory syncytial virus surveillance pilot

**DOI:** 10.1111/irv.12688

**Published:** 2019-10-31

**Authors:** Siddhivinayak Hirve, Nigel Crawford, Rakhee Palekar, Wenqing Zhang, the WHO RSV surveillance Group, Christina Bancej, Ian Barr, Elsa Baumeister, Shobha Broor, Alyeksandr Burmaa, Harry Campbell, Braulia Caetano, Mandeep Chadha, Malinee Chittaganpitch, Daouda Coulibaly, Badarch Darmaa, Joanna Ellis, Manal Fahim, Rodrigo Fasce, Kadjo Herve, Sandra Jackson, Maria Pisareva, Jocelyn Moyes, Amel Naguib, Harish Nair, Richard Pebody, Varsha Potdar, Soatiana Rajatonirina, Marilda Siqueira, Peter G. Smith, Elizaveta Smorodintseva, Viviana Sotomayor, Florette Treurnicht, Almiro Tivane, Marietjie Venter, Niteen Wairagkar, Maria Zambon

**Affiliations:** ^1^ Global Influenza Programme World Health Organization Geneva Switzerland; ^2^ Royal Children's Hospital Melbourne Vic. Australia; ^3^ Pan American Health Organization Washington DC USA

**Keywords:** case definition, respiratory syncytial virus, surveillance

## Abstract

**Background:**

The lack of a uniform surveillance case definition poses a challenge to characterize the epidemiology, clinical features, and disease burden of the respiratory syncytial virus (RSV). Global standards for RSV surveillance will inform immunization policy when RSV vaccines become available.

**Methods:**

The WHO RSV surveillance pilot leverages the capacities of the Global Influenza Surveillance and Response System (GISRS). Hospitalized and non‐hospitalized medically attended patients of any age were tested for RSV using standardized molecular diagnostics throughout the year in fourteen countries. An extended severe acute respiratory infection (extended SARI) or an acute respiratory infection (ARI) case definition was used that did not require fever as a criterion.

**Results:**

Amongst 21 221 patients tested for RSV between January 2017 and September 2018, 15 428 (73%) were hospital admissions. Amongst hospitalized RSV‐positive patients, 50% were aged <6 months and 88% <2 years. The percentage of patients testing positive for RSV was 37% in children <6 months and 25% in those aged 6 months to 2 years. Patients with fever were less likely to be RSV positive compared to those without fever (OR 0.74; 95% CI: 0.63‐0.86). For infants <6 months, 29% of RSV ARI cases did not have fever.

**Conclusion:**

Requiring fever in a case definition for RSV lowers the sensitivity to detect cases in young children. Countries should consider ways to leverage the GISRS platform to implement RSV surveillance with an augmented case definition amongst the young pediatric population.

## 1 INTRODUCTION

Globally, RSV is the leading infectious cause of respiratory morbidity and mortality in children aged <5 years. Annually, there are an estimated 33 (uncertainty range 21‐50) million episodes of RSV‐associated acute lower respiratory infection (ALRI), 3.2 (uncertainty range 2.7‐3.8) million RSV hospitalizations, and 59 600 (uncertainty range 48 000‐74 500) in‐hospital RSV deaths.^1^, ^2^ RSV vaccine research and development efforts have progressed significantly in recent years, with some nineteen vaccines and two new generation monoclonal antibody candidates in various stages of clinical trials. It is possible that a maternal RSV vaccine to prevent RSV infection in young infants may be licensed in the next few years.^3^

Though RSV disease occurs across all ages,^4^, ^5^, ^6^ it disproportionately affects children <2 years.^7^ The WHO Strategic Advisory Group of Experts on Immunization (SAGE) has recommended improved case definitions, surveillance, and disease estimates for RSV especially in low‐ and middle‐income countries where the burden is likely to be high.^8^ There is a need for improved RSV surveillance to better understand seasonality and disease burden in different countries.^9^ RSV surveillance is often a by‐product of influenza‐like illness (ILI) or severe acute respiratory infection (SARI) surveillance.^10^, ^11^ However, these case definitions require a history of fever or measured fever and consequently may miss up to a significant proportion of RSV infections especially in younger children.^12^, ^13^, ^14^ In 2016, WHO piloted a RSV surveillance strategy that leverages the capacities of the Global Influenza Surveillance and Response System (GISRS) using a broadened case definition.^15^ It aimed to establish laboratory and epidemiological standards for RSV detection to improve the understanding of seasonality, disease burden, and age‐groups at highest risk. Using the data collected in this pilot study, this paper aims to describe the clinical predictors for RSV presentation and evaluate the performance of the extended SARI and ARI case definitions for RSV surveillance.

## 2 METHODS

Surveillance sites from fourteen countries, from all six WHO regions, participated in the pilot study. Countries were selected based on having a WHO‐designated National Influenza Centre and/or a national public health laboratory, a strong national influenza surveillance system and an interest in participation in the pilot. Countries were required to test 1000 patients annually for RSV (250 patients in each of the four age groups—<6 months, 6 months to <5 years, 5 to <65 years, and 65 years and more).

### 2.1 Site profile and surveillance practices

In Argentina, Brazil, Chile, Egypt, Russian Federation, and South Africa, patients admitted to sentinel hospitals were screened across all ages, whereas in Australia and Canada pediatric hospital admissions only were screened. In Côte d'Ivoire, India, Mongolia, Mozambique, and Thailand, patients were screened across all ages in both sentinel hospitals and outpatient clinics. In the United Kingdom, patients were screened across all ages attending sentinel General Practitioner clinics, as well as children under 5 years in sentinel hospitals in England (Table 1). Canada reported data only for those patients who tested RSV positive and was excluded from the analysis. The number and type of sentinel hospitals (secondary and tertiary‐level care) varied across countries. The selection of sentinel hospitals and clinics was largely based on patient load and convenience, and there was no requirement for them to be nationally representative.

**Table 1:** Sentinel site profile, WHO RSV surveillance, 2017‐18

	Age‐group under surveillance	Patients under surveillance	# sentinel sites	Start of surveillance	No. tested (% hospitalized)
Argentina	All ages	Inpatient	6	2016 wk52	1214 (99%)
Australia	0‐18 y	Inpatient	1	2017 wk31	1560 (100%)
Brazil	All ages	Inpatient	2	2017 wk03	727 (91%)
Canada	0‐16 y	Inpatient	12	2017 wk34	2178^a^ (100%)
Chile	All ages	Inpatient	2	2017 wk01	883 (100%)
Côte d'Ivoire	All ages	Inpatient + Outpatient	9	2017 wk01	1772 (36%)
Egypt	All ages	Inpatient	4	2017 wk01	1194 (100%)
India	All ages	Inpatient + Outpatient	11	2017 wk01	1537 (82%)
Mongolia	All ages	Inpatient + Outpatient	7	2017 wk02	1175 (89%)
Mozambique	All ages	Inpatient + Outpatient	4	2017 wk01	969 (76%)
Russian Federation	All ages	Inpatient	18	2016 wk52	1648 (100%)
South Africa	All ages	Inpatient	5	2017 wk01	3409 (100%)
Thailand	All ages	Inpatient + Outpatient	11	2017 wk01	2752 (53%)
United Kingdom^b^	Pediatric	Inpatient	6	2017 wk39	2381^c^ (0%)
	All ages	Outpatient	70 GPs		

Abbreviations: GP, general practitioners; wk, weeks; y, years.

a Canada has reported data for laboratory‐confirmed RSV‐positive cases only from 11 of its 12 sentinel sites.

b Surveillance is restricted to England only.

c The United Kingdom has reported case‐based data for outpatient surveillance only.

Physicians and nurses screened patients admitted the previous day with acute onset cough or shortness of breath or, for patients attending outpatient clinics, with at least one of cough, sore throat, shortness of breath, or runny nose. Sepsis and apnea were also criteria for enrollment in infants <6 months (Table 2). Additionally, information on fever, wheeze, and associated risk factors, such as prematurity, malnutrition, cardiac and respiratory illness, and immunodeficiency, were recorded on a standardized case record form. The sampling strategy varied and ranged from screening of all eligible patients to screening a sample of eligible patients (eg, on certain days of the week). Patients were screened all year‐round except in Canada and England where patients were screened between November and June. Age‐appropriate nasal, nasopharyngeal, or lower respiratory tract specimens were collected and transported in virus transport media for laboratory RSV testing.^16^ Specimens from patients with reported or measured fever were additionally tested for influenza and subtyped using standardized rRT‐PCR assays.^17^

**Table 2:** Surveillance case definitions for RSV, 2017‐18

	RSV	Influenza
In patient	Extended SARI Severe (overnight hospitalization) Acute (onset within past 10 d) Respiratory infection (cough or shortness of breath) In infants <6 mo age Apnea^a^ Sepsis Fever more than 37.5°C or hypothermia Shock^b^ Seriously ill without apparent cause	SARI Severe (overnight hospitalization) Acute (onset within past 10 d) History of fever or measured fever of 38°C or more Respiratory infection (cough or shortness of breath)
Out patient	ARI Acute (onset within past 10 d) Respiratory infection (at least one of cough, sore throat, shortness of breath or runny nose) Extended ILI Acute (onset within past 10 d) Respiratory infection (cough)	ILI Onset within past 10 d Measured fever of 38°C or more, and Cough

Abbreviations: ARI, acute respiratory infection; ILI, influenza‐like illness; RSV, respiratory syncytial virus; SARI, severe acute respiratory infection.

a Apnea defined as temporary cessation of breathing from any cause.

b Shock defined as lethargy, fast breathing, cold skin, prolonged capillary refill or weak pulse.

### 2.2 Laboratory testing

All laboratories participated in an external quality assurance program using proficiency panels developed by the Division of Viral Diseases at the US Centers for Disease Control and Prevention (CDC), consisting of contemporary and historical strains of RSV‐A and RSV‐B or panels supplied by Quality Control for Molecular Diagnostics (QCMD), United Kingdom. Specimens received by laboratories were stored at −70°C and batch tested using a standardized rRT‐PCR assay developed by CDC for generic RSV detection. The CDC molecular assay was compatible with a wide range of virus transport media and different extraction systems and PCR amplification platforms. Primers and probes were supplied by CDC, and extraction reagent kits and amplification enzymes were supplied by International Reagent Resource (IRR) of CDC. National laboratories had the option to use commercial or in‐house laboratory developed tests (LDTs) provided these were validated against the CDC RSV assay. The US CDC, Public Health England, Colindale, and National Institute of Communicable Diseases, Johannesburg provided training and quality assurance support, as required, to all participating national laboratories. The WHO FluMart data platform was adapted to receive case‐based clinical and laboratory RSV data, and countries were required to upload anonymized RSV surveillance data every week or every fortnight. Public access to an interactive aggregated RSV surveillance output was provided at http://ais.paho.org/phip/viz/ed_who_rsv.asp.

### 2.3 Data analysis

Data from Canada were excluded for analysis as they had reported only on RSV‐positive cases. The start of surveillance varied (range epidemiological week (EW) 52 of 2016 to EW 39 of 2017) with 11 of 14 countries initiating surveillance in the first quarter of 2017. The end date for this analysis was set at EW 39 of 2018. England reported data only from their General Practitioner sentinel surveillance (Table 1). All analyses were done separately for inpatient and outpatient surveillance and disaggregated into six age‐groups—<6 months, 6 months to <2 years, 2 to <5 years, 5 to <18 years, 18 to <65 years, and 65 years and more. Univariate logistic regression was used to determine clinical predictors for RSV cases. The SARI and ILI case definitions were evaluated against the extended SARI and ARI definitions that did not require fever. The relative sensitivity was estimated as it was not known how many RSV cases were missed who did not fit the extended SARI or ARI case definitions. The positive predictive value (PPV) was calculated for the age‐specific percent positivity cumulated over the study period. We also evaluated the individual performance of apnea and sepsis (for infants <6 months), and when wheezing (clinical presentation of RSV‐associated bronchiolitis) was added to the extended SARI case definition. The area under the curve (AUC) was estimated as a measure of accuracy of how well an alternate case definition separates the group being tested into those with and without the disease as determined by the existing reference case definition. An AUC (range 0‐1) <0.7 indicates a poor discriminatory ability of the alternate case definition in relation to the reference case definition. All analysis was done with Stata v15 software.

## 3 RESULTS

**Figure 1:**
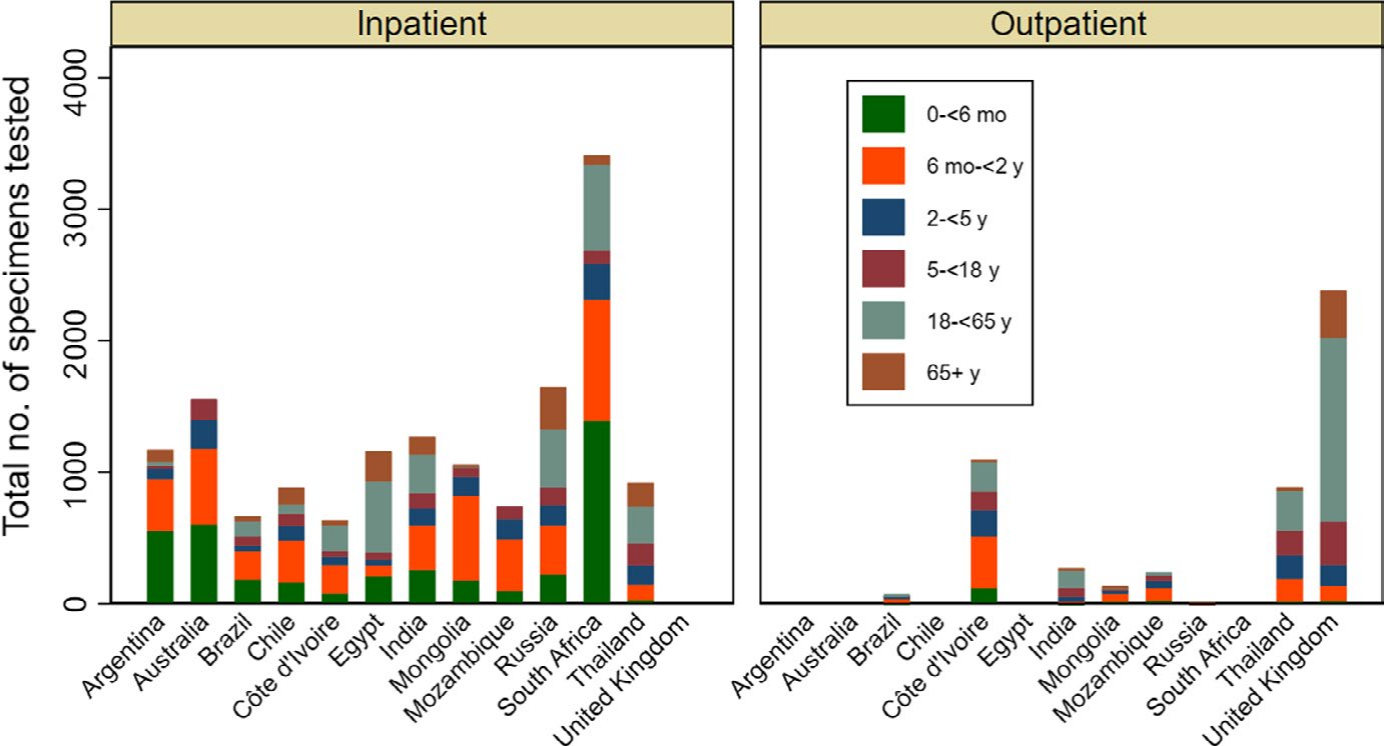
Age distribution of specimens tested, WHO RSV surveillance, 2017‐18

A total of 21 221 patients (excluding Canada) were tested for RSV of whom 15 428 (73%) were inpatients (Table 1). Overall, 60% of patients tested were aged <5 years (20% <6 months, 30% between 6 months to 2 years and 10% between 2 and 5 years). The age distribution of patients tested varied by country (Figure 1). Overall, <10% of patients tested were aged 65 years or older (Table 3). Amongst inpatients, a total of 2963 (19.2%) tested positive for RSV. Amongst RSV‐positive hospitalized patients, 2598 (87.6%) were <2 years age, and 1461 (49.3%) were <6 months. The percentage testing positive for RSV was similar in males and females.

**Table 3:** Age, sex distribution of patients, and RSV detection, 2017‐18

	Inpatient surveillance		Tested n (%)	Outpatient surveillance		Tested n (%)
	RSV negative n (%)	RSV positive n (%)		RSV negative n (%)	RSV positive n (%)	
Age						
0 to <6 m	2493 (20.5)	1461 (49.3)	3954 (26.2)	157 (3.3)	40 (9.9)	197 (3.9)
6 m to <2 y	3456 (28.5)	1137 (38.3)	4593 (30.4)	726 (15.6)	157 (38.9)	883 (17.5)
2 to <5 y	1365 (11.2)	187 (6.3)	1552 (10.3)	579 (12.5)	93 (23.0)	672 (13.3)
5 to <18 y	1077 (8.8)	49 (1.6)	1126 (7.4)	768 (16.6)	19 (4.7)	787 (15.6)
18 to <65 y	2546 (21.0)	74 (2.5)	2620 (17.3)	1991 (43.0)	74 (18.8)	2065 (41.0)
65+ y	1175 (9.7)	55 (1.8)	1230 (8.1)	405 (8.7)	20 (4.9)	425 (8.4)
Male	6255 (53.3)	1565 (53.9)	7820 (53.4)	1241 (52.7)	160 (57.5)	1401 (53.2)

### 3.1 Clinical characteristics

**Figure 2:**
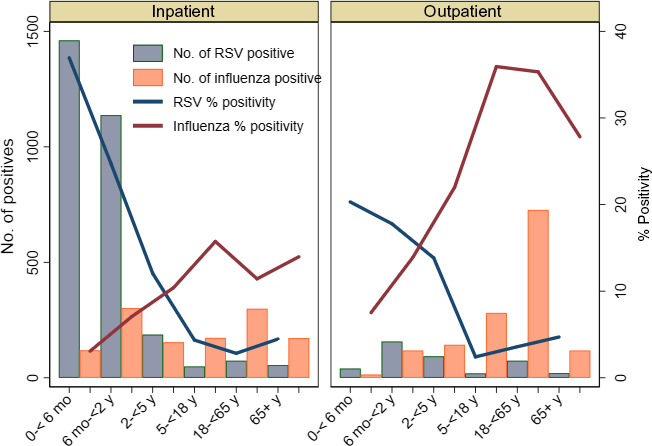
Age distribution and percent positivity of RSV and influenza, 2017‐18

**Figure 3:**
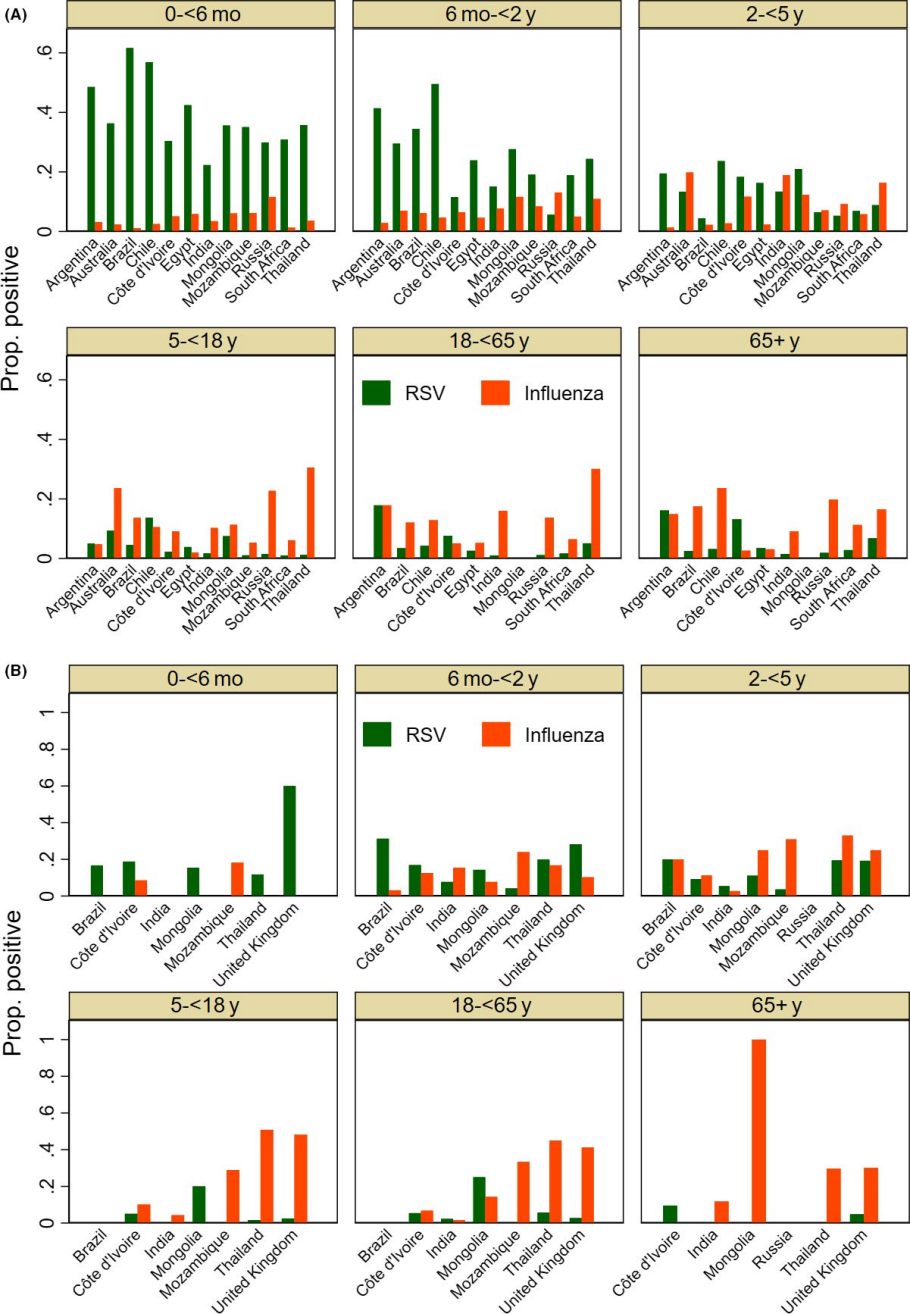
A, RSV and influenza proportion positive (inpatient surveillance), 2017‐18. B, RSV and influenza proportion positive (outpatient surveillance), 2017‐18

Amongst hospitalized patients, RSV percent positivity was highest (37%) in infants <6 months, 25% in children 6 months to 2 years, and 12% in children 2‐5 years age. RSV percent positivity was about 4% in adults and older adults aged 65 years and more. RSV percent positivity was lower but showed similar age trends amongst non‐hospitalized patients. In contrast, percent positivity for influenza amongst hospitalized patients was 3%‐7% in children <2 years, 11% in adults aged 18 to <65 years, and 14% in older adults aged over 65 years (Figure 2). The age‐group stratified RSV percent positivity varied across countries. RSV predominated over influenza in children <2 years age (especially infants <6 months) whereas the reverse was seen in older adults aged 65 years and more. This trend was seen for both hospitalized and non‐hospitalized patients across all the participating countries (Figure 3).

Amongst hospitalized patients <6 months, apnea was significantly more prevalent amongst RSV‐positive infants (8.3%) than those who tested negative (6.0%) (odds ratio (OR) 1.42; 95% CI: 1.05‐1.94) (Table 4). Sepsis was less common amongst RSV‐positive infants (4.7%) than those who tested negative (10%) (OR ‐ 0.44; 95% CI: 0.31‐0.64). Cough (OR: 6.21, 95% CI: 4.77‐8.08), shortness of breath (OR: 1.64, 95% CI: 1.38‐1.96), wheezing (OR: 1.73, 95% CI: 1.49‐2.01), runny nose (OR: 1.75, 95% CI: 1.50‐2.04), and lower chest in‐drawing (OR: 2.46, 95% CI: 2.07‐2.91) were all significantly more common in RSV‐infected infants. The findings were similar for hospitalized children aged 6 months to 2 years, though the association with wheeze was not significant.

**Table 4:** Clinical predictors for laboratory‐confirmed RSV for patients with extended SARI and ARI case definition, 2017‐18

	Inpatient surveillance		Univariate Odds ratio (95% CI)	Outpatient surveillance		Univariate Odds ratio (95% CI)
	RSV negative n (%)	RSV positive n (%)		RSV negative n (%)	RSV positive n (%)	
Young infants (0 to <6 m)						
Apnea	103 (6.0)	75 (8.3)	1.42 (1.05‐1.94)	0 (0)	0 (0)	–
Sepsis	166 (10.0)	37 (4.7)	0.44 (0.31‐0.64)	0 (0)	0 (0)	–
Cough	1813 (76.3)	1343 (95.2)	6.21 (4.77‐8.08)	147 (93.6)	37 (92.5)	0.83 (0.21‐3.20)
Shortness of breath	643 (49.3)	531 (61.6)	1.64 (1.38‐1.96)	17 (11.2)	9 (28.1)	3.08 (1.22‐7.74)
Fever (history)	1044 (53.4)	503 (45.9)	0.74 (0.63‐0.86)	127 (81.9)	26 (76.4)	0.71 (0.29‐1.74)
Fever (≥38°C)	1718 (76.8)	847 (68.8)	0.66 (0.57‐0.77)	19 (38.7)	4 (80.0)	6.31 (0.65‐60.8)
Wheeze	669 (32.0)	527 (45.0)	1.73 (1.49‐2.01)	2 (1.5)	3 (10)	6.94 (1.10‐43.5)
Runny nose	856 (44.1)	590 (58.0)	1.75 (1.50‐2.04)	2 (5.8)	2 (66.6)	32.0 (1.95‐522.7)
Lower chest in‐drawing	717 (41.5)	525 (63.6)	2.46 (2.07‐2.91)	0 (0)	1 (50)	–
Pre‐existing condition^a^	453 (18.1)	190 (7.9)	0.67 (0.56‐0.80)	6 (4.0)	0 (0)	–
Children (6 m to <2 y)						
Cough	3170 (93.5)	1066 (96.5)	1.92 (1.35‐2.74)	656 (91.2)	140 (90.3)	0.89 (0.49‐1.62)
Shortness of breath	1483 (57.9)	565 (65.7)	1.39 (1.18‐1.63)	92 (13.8)	27 (20.3)	1.58 (0.98‐2.55)
Fever (history)	1858 (65.6)	597 (61.7)	0.84 (0.72‐0.98)	583 (82.3)	126 (82.3)	1.00 (0.63‐1.58)
Fever (≥38°C)	2188 (71.6)	691 (69.3)	0.89 (0.76‐1.04)	162 (54.9)	37 (77.0)	2.76 (1.35‐5.62)
Wheeze	1102 (38.7)	384 (41.1)	1.10 (0.94‐1.28)	38 (6.6)	14 (10.6)	1.67 (0.87‐3.18)
Sore throat	629 (27.6)	218 (32.2)	1.24 (1.03‐1.49)	101 (15.7)	21 (16.9)	1.09 (0.65‐1.82)
Runny nose	1167 (49.4)	472 (61.3)	1.61 (1.36‐1.90)	22 (13.9)	3 (21.4)	1.68 (0.43‐6.52)
Lower chest in‐drawing	904 (42.4)	343 (56.4)	1.75 (1.46‐2.10)	6 (9.0)	3 (30.0)	4.28 (0.87‐21.0)
Pre‐existing condition^a^	590 (17.0)	183 (9.5)	0.93 (0.77‐1.11)	17 (2.6)	6 (4.8)	1.86 (0.72‐4.83)
Children (2 to <5 y)						
Cough	1250 (92.8)	172 (93.4)	1.10 (0.59‐2.04)	542 (95.0)	86 (96.6)	1.48 (0.44‐4.97)
Shortness of breath	586 (54.3)	90 (54.2)	0.99 (0.71‐1.38)	57 (12.0)	14 (19.4)	1.75 (0.92‐3.35)
Fever (history)	736 (64.7)	98 (60.4)	0.83 (0.59‐1.16)	459 (81.2)	77 (83.7)	1.18 (0.65‐2.14)
Fever (≥38°C)	940 (75.5)	121 (71.6)	0.81 (0.57‐1.17)	182 (70.5)	36 (85.7)	2.50 (1.01‐6.19)
Wheeze	386 (34.0)	60 (36.5)	1.11 (0.79‐1.56)	33 (7.8)	10 (14.0)	1.92 (0.90‐4.09)
Sore throat	303 (31.8)	43 (34.9)	1.15 (0.77‐1.70)	123 (27.1)	25 (39.6)	1.76 (1.02‐3.04)
Runny nose	414 (48.3)	60 (50.8)	1.10 (0.75‐1.62)	16 (14.2)	2 (28.5)	2.40 (0.42‐13.4)
Lower chest in‐drawing	277 (36.8)	47 (45.6)	1.43 (0.95‐2.17)	8 (13.7)	0 (0)	–
Pre‐existing condition^a^	305 (22.3)	33 (9.5)	0.74 (0.50‐1.10)	23 (5.0)	3 (4.7)	0.93 (0.27‐3.20)
Children (5 to <18 y)						
Cough	929 (86.7)	45 (91.8)	1.71 (0.60‐4.85)	693 (96.1)	16 (84.2)	0.21 (0.05‐0.78)
Shortness of breath	471 (44.3)	24 (48.9)	1.20 (0.67‐2.13)	87 (17.1)	4 (30.7)	2.14 (0.64‐7.10)
Fever (history)	600 (66.0)	21 (45.6)	0.43 (0.23‐0.78)	635 (85.4)	17 (89.4)	1.44 (0.32‐6.34)
Fever (≥38°C)	669 (69.6)	27 (62.7)	0.73 (0.39‐1.38)	237 (77.2)	4 (100.0)	–
Wheeze	178 (18.9)	9 (19.5)	1.04 (0.49‐2.19)	30 (6.9)	1 (8.3)	1.20 (0.15‐9.68)
Sore throat	350 (37.6)	8 (23.5)	0.50 (0.22‐1.13)	218 (48.0)	2 (18.1)	0.24 (0.05‐1.12)
Runny nose	197 (36.4)	15 (57.6)	2.38 (1.07‐5.28)	21 (16.6)	0 (0)	–
Lower chest in‐drawing	95 (22.8)	13 (52.0)	3.66 (1.61‐8.28)	0 (0)	1 (100)	–
Pre‐existing condition^a^	158 (14.6)	13 (8.6)	2.10 (1.08‐4.04)	21 (4.6)	1 (9.0)	2.06 (0.25‐16.8)
Adults (18 to <65 y)						
Cough	2326 (92.3)	71 (95.9)	1.95 (0.60‐6.26)	1746 (96.2)	64 (94.1)	0.63 (0.22‐1.78)
Shortness of breath	1351 (53.8)	39 (54.1)	1.01 (0.63‐1.62)	572 (50.6)	21 (41.1)	0.68 (0.38‐1.20)
Fever (history)	1493 (84.4)	43 (79.6)	0.72 (0.36‐1.41)	1592 (88.8)	53 (80.3)	0.51 (0.27‐0.95)
Fever (≥38°C)	2051 (94.3)	47 (85.4)	0.35 (0.16‐0.76)	353 (83.2)	20 (83.3)	1.00 (0.33‐3.03)
Wheeze	432 (17.8)	9 (12.5)	0.65 (0.32‐1.33)	184 (22.8)	7 (16.6)	0.67 (0.29‐1.54)
Sore throat	799 (41.7)	20 (35.0)	0.75 (0.43‐1.31)	387 (61.4)	17 (48.5)	0.59 (0.29‐1.17)
Runny nose	388 (40.6)	10 (52.6)	1.62 (0.65‐4.02)	43 (29.0)	2 (28.5)	0.97 (0.18‐5.22)
Lower chest in‐drawing	70 (12.1)	2 (28.5)	2.90 (0.55‐15.2)	0 (0)	1 (16.6)	–
Pregnant	148 (9.0)	1 (2.3)	0.23 (0.03‐1.75)	12 (2.5)	2 (8.0)	3.34 (0.70‐15.8)
Pre‐existing condition^b^	860 (33.7)	31 (41.8)	1.41 (0.88‐2.25)	61 (9.5)	3 (8.3)	0.86 (0.25‐2.89)
Older adults (65+ y)						
Cough	1065 (92.1)	51 (92.7)	1.08 (0.38‐3.08)	359 (97.8)	17 (94.4)	0.37 (0.04‐3.20)
Shortness of breath	763 (66.1)	39 (70.9)	1.24 (0.68‐2.25)	146 (74.4)	9 (75.0)	1.02 (0.26‐3.94)
Fever (history)	423 (62.2)	24 (58.5)	0.85 (0.45‐1.62)	308 (94.4)	16 (100)	–
Fever (≥38°C)	896 (83.8)	36 (75.0)	0.57 (0.29‐1.13)	34 (82.9)	0 (0)	–
Wheeze	298 (27.2)	22 (40.0)	1.78 (1.02‐3.10)	54 (47.7)	2 (40.0)	0.72 (0.11‐4.52)
Sore throat	242 (29.9)	10 (24.3)	0.75 (0.36‐1.56)	35 (58.3)	1 (33.3)	0.35 (0.03‐4.15)
Runny nose	107 (38.6)	7 (36.8)	0.92 (0.35‐2.42)	7 (36.8)	0 (0)	–
Lower chest in‐drawing	62 (19.8)	6 (30.0)	1.72 (0.63‐4.67)	0 (0)	0 (0)	–
Pre‐existing condition^b^	762 (64.8)	37 (67.2)	1.11 (0.62‐1.98)	21 (31.8)	1 (33.3)	1.07 (0.09‐12.4)

a Pre‐existing condition includes prematurity, chronic respiratory disease including asthma, malnutrition, or immunodeficiency.

b Pre‐existing condition includes respiratory disease, diabetes, heart disease, or immunodeficiency.

The presence of either reported or measured fever was significantly less likely amongst RSV cases than RSV‐negative children <2 years than in those aged 6 months to 2 years. Similar trends in clinical predictors were seen in older children, adults, and older adults but were generally not statistically significant. Presence of a pre‐existing illness was not significantly associated with RSV infection except in children aged 5‐18 years. Clear statistically significant trends for RSV infection for most clinical predictors could not be ascertained for patients for any of the age‐groups attending outpatient clinics in a primary care setting, but these analyses were based on smaller numbers of patients and the confidence intervals on odds ratio estimates were generally wide.

### 3.2 Case definition

Amongst hospitalized infants <6 months age, 29% of those RSV cases that otherwise had been captured by an extended SARI case definition were missed after inclusion of measured or reported fever. The positive predictive value (PPV) of the SARI case definition that included fever was 34% for infants <6 months and 24% for children between 6 months to <2 years. The ability of fever to correctly identify the presence or absence of RSV infection was poor (area under the curve (AUC) – 0.31). The percent of missed cases was lower (18‐20%) in children 6 months to <5 years age. Apnea and sepsis individually had low sensitivity (8.3% and 4.7%) but were highly specific (93% and 89%) for RSV infection in infants <6 months. The addition of wheeze to the extended SARI case definition reduced the sensitivity to 36% in hospitalized infants <6 months age and to about 32‐33% in children aged 6 months to 5 years. Similar trends were seen amongst non‐hospitalized children, albeit with lower certainty. Amongst hospitalized older adults aged 65 years and more, the sensitivity of the SARI case definition after inclusion of fever and wheeze was 78% and 40%, respectively. Amongst non‐hospitalized older adults aged 65 years and more, the addition of fever reduced the sensitivity to 27%. However, the addition of wheeze to the extended SARI case definition increased the sensitivity to 98%, respectively (Table 5).

**Table 5:** Performance of SARI and ILI case definitions with reference to extended SARI (inpatient surveillance) and ARI (outpatient surveillance), 2017‐18

	Inpatient surveillance		PPV (95% CI)	AUC	Outpatient surveillance		PPV (95% CI)	AUC
	Sensitivity (95% CI)	Specificity (95% CI)			Sensitivity (95% CI)	Specificity (95% CI)		
Young infants (0 to <6 m)	Percent positivity – 36.9%				Percent positivity – 15.8%			
SARI (inpatient)/ILI (outpatient)	71% (69‐73)	20% (18 −21)	34% (32‐36)	0.31	71% (51‐86)	15% (10‐22)	13% (8‐20)	0.43
Apnea	8.3% (6‐10)	93% (92‐95)	42% (34‐49)	0.51	–	–	–	
Sepsis	4.7% (3‐6)	89% (88‐91)	18% (13‐24)	0.47	–	–	–	
Ex‐SARI/ARI + wheeze	36% (33‐38)	73% (71‐74)	44% (41‐46)	0.47	0% (2‐12)	98% (95‐99)	–	0.49
Children (6 m to <2 y)	Percent positivity – 24.7%				Percent positivity – 16.1%			
SARI (inpatient)/ILI (outpatient)	82% (80‐84)	16% (15‐17)	24% (23‐26)	0.32	83% (76‐89)	11% (8‐13)	15% (12‐18)	0.47
Ex‐SARI/ARI + wheeze	33% (31‐36)	68% (66‐69)	25% (23‐28)	0.44	1.6% (0.2‐6)	97% (96‐98)	12% (2‐39)	0.49
Children (2 to <5 y)	Percent positivity – 12.0%				Percent positivity −12.2%			
SARI (inpatient)/ILI (outpatient)	80% (74‐85)	16% (14‐18)	11% (9‐13)	0.29	92% (82‐97)	9.9% (7‐13)	12% (9‐15)	0.51
Ex‐SARI/ARI + wheeze	32% (25‐39)	71% (69‐74)	13% (10‐17)	0.44	0% (0‐6)	97% (95‐98)	–	0.48
Children (5 to <18 y)	Percent positivity – 4.3%				Percent positivity – 2.3%			
SARI (inpatient)/ILI (outpatient)	77% (63‐87)	18% (15‐20)	4% (2‐5)	0.21	81% (48‐97)	8.3% (6‐11)	2% (1‐4)	0.45
Ex‐SARI/ARI + wheeze	18% (9‐32)	83% (81‐85)	4% (2‐9)	0.44	0% (0‐28)	99% (98‐100)	–	0.49
Adults (18 to <65 y)	Percent positivity – 2.8%				Percent positivity – 5.3%			
SARI (inpatient)/ILI (outpatient)	89% (79‐94)	6.6% (5‐7)	2% (2‐3)	0.47	86% (70‐95)	16% (13‐19)	5% (3‐7)	0.51
Ex‐SARI/ARI + wheeze	12% (6‐22)	83% (81‐84)	2% (0.9‐4)	0.47	2.7% (0.1‐14)	98% (97‐99)	9% (0.4‐42)	0.50
Older adults (65+ y)	Percent positivity – 4.4%				Percent positivity – 4.3%			
SARI (inpatient)/ILI (outpatient)	78% (64‐87)	15% (13‐17)	4% (3‐5)	0.46	66% (9‐99)	27% (17‐39)	4% (0.6‐14)	0.46
Ex‐SARI/ARI + wheeze	40% (27‐54)	74% (72‐77)	6% (4‐10)	0.57	0% (0‐70)	98% (91‐99)	–	0.49

Abbreviations: ARI, acute respiratory infection; AUC, area under curve; CI, confidence interval; Ex‐SARI, extended severe acute respiratory infection; ILI, influenza‐like illness; PPV, positive predictive value; SARI, severe acute respiratory infection.

## 4 DISCUSSION

The lack of a global uniform surveillance case definition for RSV complicates the interpretation of surveillance data. In the WHO RSV surveillance pilot, the use of an extended SARI or an ARI case definition substantially increased the number of RSV infections detected also seen in other studies.^12^, ^13^ These definitions do not require fever to identify a suspect case. On the other hand, the inclusion of fever in the RSV surveillance case definition may not matter if the objective is solely to ascertain onset of the RSV season. However, including fever may significantly compromise the use of surveillance data to estimate RSV disease burden.

Surveillance confirmed the high burden of RSV in children <2 years, especially in infants <6 months.^7^, ^18^, ^19^, ^20^ RSV commonly manifests clinically in infants with bronchiolitis.^21^, ^22^, ^23^ Wheeze has been one of the clinical end points of interest to evaluate vaccine efficacy trials.^24^ From a surveillance case definition perspective, including wheeze as a criteria reduces its sensitivity in children.^25^ Apnea, though it lacked sensitivity, was a significant clinical predictor for severe RSV infection requiring hospitalization in infants <6 months.^22^, ^26^, ^27^, ^28^ The reason for sepsis to be significantly less common amongst RSV‐positive hospitalized young infants in this study in contrast to other studies 29, 30 is unclear.

The RSV surveillance case definition is not intended to modify or replace the SARI or ILI case definition for influenza surveillance. Countries reported challenges in the adoption of the extended SARI case definition in the early stages of the RSV surveillance which were resolved through training. In practice, the physician or nurse in a sentinel hospital engaged in both RSV and influenza surveillance, screened patients with acute onset cough or shortness of breath, and collected an appropriate respiratory specimen. Information on the presence or absence of fever was recorded in the specimen requisition form and sent to the laboratory along with the respiratory specimen. At the laboratory, all the specimens were tested for RSV, whereas those specimens from patients with fever were additionally tested for influenza. Moreover, the results for influenza were reported to FluNet only for those patients with fever. Notwithstanding the additional burden of reporting, this ensured that the RSV surveillance did not disturb the influenza surveillance system but complemented it by targeting a very young age‐group that is important, yet often underrepresented in influenza surveillance.

The WHO RSV surveillance pilot had several limitations, and its early findings need to be interpreted with some caveats. Generalization of the findings should be made with caution. First, the number, type, and selection of sentinel sites varied across countries and were generally not designed to be nationally representative or comparable in terms of the clinical severity of patients included. Second, the strategy for sampling patients for testing varied across sentinel sites. This would need to be accounted for when estimating disease burden from surveillance data but is of no consequence for this analysis. Third, Brazil faced a shortfall of extended SARI cases and had to bridge the gap with patients sourced from SARI surveillance. However, with over 20 000 patients from all countries pooled together, the bias in evaluating the performance of fever as a criterion for detecting RSV would be small. Fourth, the PPV is likely to be higher during the RSV season, but it was beyond the scope of this analysis to robustly determine country‐specific seasonality patterns from data that covered just 2 years.

There were several factors that could cause potential bias in the study. The clinical data were collected from patients or caregivers or physician records and could be subject to recall or measurement bias. It was beyond the scope of this analysis to evaluate the influence of recall of symptoms based on the interval between the onset of symptoms and when patients presented at the hospital or clinic. On the other hand, recall bias is not expected to be related to the RSV infection status. The health infrastructure, and healthcare availability, affordability and accessibility varied across different country contexts. The health seeking behavior of populations is likely to vary across different cultural contexts. The hospital admission practices are likely to vary across different settings as indicated by the heterogeneity in the severity of illness of patients screened for RSV also varied across different sites. It is beyond the scope, and not the purpose, of the RSV or any routine national disease surveillance system to collect information on these variables which are better studied in specialized research settings.

In conclusion, we found that pilot countries with existing influenza surveillance were able to build on RSV surveillance on top of their influenza surveillance program with marginal incremental costs and effort. Countries did not report any significant adverse impact on influenza surveillance. The inclusion of fever as a criterion substantially reduces the sensitivity of a case definition to detect RSV infection in young children, where the burden of RSV is the highest. WHO should continue to closely monitor for adverse impact, if any, on influenza surveillance in different settings.

## APPENDIX A

### WHO RSV SURVEILLANCE GROUP

Christina Bancej^1^, Ian Barr^2^, Elsa Baumeister^3^, Shobha Broor^4^, Alyeksandr Burmaa^5^, Harry Campbell^6^, Braulia Caetano^7^, Mandeep Chadha^8^, Malinee Chittaganpitch^9^, Daouda Coulibaly^10^, Badarch Darmaa^11^, Joanna Ellis^12^, Manal Fahim^13^, Rodrigo Fasce^14^, Kadjo Herve^15^, Sandra Jackson^16^, Maria Pisareva^17^, Jocelyn Moyes^18^, Amel Naguib^19^, Harish Nair^6^, Richard Pebody^20^, Varsha Potdar^8^, Soatiana Rajatonirina^21^, Marilda Siqueira^7^, Peter G. Smith^22^, Elizaveta Smorodintseva^23^, Viviana Sotomayor^24^, Florette Treurnicht^18^, Almiro Tivane^25^, Marietjie Venter^26^, Niteen Wairagkar^27^, Maria Zambon^12^

^1^Centre for Immunization and Respiratory Infections, Public Health Agency of Canada, Ottawa, Canada

^2^Victorian Infectious Diseases Reference Laboratory, Peter Doherty Institute for Infection and Immunity, Melbourne, Australia

^3^Departamento Virologia, INEI‐ANLIS “Carlos G Malbrán”, Buenos Aires, Argentina

^4^Medicine and Health Sciences, Shree Guru Gobind Singh Tricentenary University, Gurugram, India

^5^National Influenza Surveillance Division, National Center for Communicable Diseases, Ministry of Health, Ulaanbaatar, Mongolia

^6^Usher Institute of Population Health Research and Informatics, University of Edinburgh, Edinburgh, United Kingdom

^7^Institute Oswaldo Cruz/FIOCRUZ, Rio de Janeiro, Brazil

^8^National Institute of Virology, Indian Council of Medical Research, Pune, India

^9^Department of Medical Sciences, Ministry of Public Health, Nonthaburi, Thailand

^10^Epidemiological Surveillance and Research, Institut National de l'Hygiène Publique, Abidjan, Côte d'Ivoire

^11^Virology Laboratory, National Center for Communicable Diseases, Ulaanbaatar, Mongolia

^12^Virus Reference Department, Public Health England, London, United Kingdom

^13^Dept of Surveillance and Epidemiology, Ministry of Health and Population, Cairo, Egypt

^14^Sub‐department of Viral Diseases, Instituto de Salud Pública de Chile, Santiago, Chile

^15^Department of Epidemic Viruses, Institut Pasteur de Côte d'Ivoire, Abidjan, Côte d'Ivoire

^16^Global Influenza Program, World Health Organization, Geneva, Switzerland

^17^Laboratory of Molecular Virology, Smorodintsev Research Institute of Influenza, St. Petersburg, Russian Federation

^18^Center for Respiratory Diseases and Meningitis, National Institute for Communicable Diseases, Johannesburg, South Africa

^19^Central Public Health Laboratory, Ministry of Health, Cairo, Egypt

^20^Flu Surveillance, Public Health England, London, United Kingdom

^21^African Region Office, World Health Organization, Brazzaville, Republic of Congo

^22^MRC Tropical Epidemiology Group, London School of Hygiene and Tropical Medicine, London, United Kingdom

^23^Laboratory for studying risk factors of Influenza and ARVI, Smorodintsev Research Institute of Influenza, Ministry of Health of the Russian Federation, St. Petersburg, Russian Federation

^24^Division de Planificacion Sanitaria, Ministerio de Salud Chile, Santiago, Chile

^25^Laboratório de Isolamento Viral, Instituto Nacional de Saúde, Maputo, Mozambique

^26^Center for Viral Zoonosis, Department of Medical Virology, University of Pretoria, South Africa

^27^Bill and Melinda Gates Foundation, Seattle, United States

## References

[1] Shi T , McAllister DA , O'Brien KL , et al. Global, regional, and national disease burden estimates of acute lower respiratory infections due to respiratory syncytial virus in young children in 2015: a systematic review and modelling study. Lancet 2017;390(10098):946‐958. 28689664 10.1016/S0140-6736(17)30938-8 PMC5592248

[2] PATH The global respiratory syncytial virus burden ‐ a summary of the latest estimates. 2018.

[3] PATH . RSV vaccine and mAb snapshot. 2018 https://vaccineresources.org/details.php?i=1562. Accessed October 16, 2018.

[4] Falsey AR . Respiratory syncytial virus infection in adults. Semin Respir Crit Care Med. 2007;28(2):171‐181. 17458771 10.1055/s-2007-976489

[5] Falsey AR . Respiratory syncytial virus infection in elderly and high‐risk adults. Exp Lung Res. 2005;31(Suppl 1):77. 16395866

[6] Hall CB , Weinberg GA , Iwane MK , et al. The burden of respiratory syncytial virus infection in young children. N Engl J Med. 2009;360(6):588‐598. 19196675 10.1056/NEJMoa0804877 PMC4829966

[7] Shi T , McAllister DA , O'Brien KL , et al. Global, regional, and national disease burden estimates of acute lower respiratory infections due to respiratory syncytial virus in young children in 2015: a systematic review and modelling study. Lancet. 2017;390(10098):946‐958. 28689664 10.1016/S0140-6736(17)30938-8 PMC5592248

[8] Modjarrad K , Giersing B , Kaslow DC , Smith PG , Moorthy VS . WHO consultation on respiratory syncytial virus vaccine development report from a World Health Organization Meeting held on 23–24 March 2015. Vaccine. 2016;34(2):190‐197. 26100926 10.1016/j.vaccine.2015.05.093 PMC6858870

[9] Giersing BK , Vekemans J , Nava S , Kaslow DC , Moorthy V . Report from the World Health Organization's third Product Development for Vaccines Advisory Committee (PDVAC) meeting, Geneva, 8–10th June 2016. Vaccine. 2017:8‐10. 10.1016/j.vaccine.2016.10.090 PMC7131228 28262332

[10] Breiman RF , Van Beneden CA , Farnon EC . Surveillance for respiratory infections in low‐ and middle‐income countries: experience from the Centers for Disease Control and Prevention's Global Disease Detection International Emerging Infections Program. J Infect Dis. 2013;208(Suppl 3):S167‐S172. 24265474 10.1093/infdis/jit462 PMC7107375

[11] Haynes AK , Manangan AP , Iwane MK , et al. Respiratory syncytial virus circulation in seven countries with Global Disease Detection Regional Centers. J Infect Dis. 2013;208(Suppl 3):S246‐S254. 24265484 10.1093/infdis/jit515

[12] Rha B , Dahl RM , Moyes J , et al. Performance of surveillance case definitions in detecting respiratory syncytial virus infection among young children hospitalized with severe respiratory illness‐South Africa, 2009–2014. J Pediatric Infect Dis Soc. 2019;8(4):325‐333. 29931284 10.1093/jpids/piy055 PMC12813577

[13] Saha S , Pandey BG , Choudekar A , et al. Evaluation of case definitions for estimation of respiratory syncytial virus associated hospitalizations among children in a rural community of northern India. J Glob Health. 2015;5(2):010419. 26649172 10.7189/jogh.05.020419 PMC4652925

[14] Nyawanda BO , Mott JA , Njuguna HN , et al. Evaluation of case definitions to detect respiratory syncytial virus infection in hospitalized children below 5 years in Rural Western Kenya, 2009–2013. BMC Infect Dis. 2016;16:218. 27207342 10.1186/s12879-016-1532-0 PMC4875667

[15] World Health Organization . WHO strategy to pilot Global Respiratory Syncytial Virus surveillance based on the Global Influenza Surveillance and Response System (GISRS). 2017.

[16] World Health Organization Global Epidemiological Surveillance Standards for Influenza . 2014 http://www.who.int/influenza/resources/documents/WHO_Epidemiological_Influenza_Surveillance_Standards_2014.pdf. Accessed September 29, 2019.

[17] World Health Organization . G.I.S.N. Manual for the laboratory diagnosis and virological surveillance of influenza. 2011.

[18] Nair H , Nokes DJ , Gessner BD , et al. Global burden of acute lower respiratory infections due to respiratory syncytial virus in young children: a systematic review and meta‐analysis. Lancet. 2010;375(9725):1545‐1555. 20399493 10.1016/S0140-6736(10)60206-1 PMC2864404

[19] Nair H , Simões EAF , Rudan I , et al. Global and regional burden of hospital admissions for severe acute lower respiratory infections in young children in 2010: a systematic analysis. Lancet. 2013;381(9875):1380‐1390. 23369797 10.1016/S0140-6736(12)61901-1 PMC3986472

[20] Schanzer DL , Saboui M , Lee L , Nwosu A , Bancej C . Burden of influenza, respiratory syncytial virus, and other respiratory viruses and the completeness of respiratory viral identification among respiratory inpatients, Canada, 2003–2014. Influenza Other Respir Viruses. 2018;12(1):113‐121. 29243369 10.1111/irv.12497 PMC5818333

[21] Ghazaly M , Nadel S . Characteristics of children admitted to intensive care with acute bronchiolitis. Eur J Pediatr. 2018;177(6):913‐920. 29654399 10.1007/s00431-018-3138-6 PMC5958152

[22] Laham FR , et al. Clinical profiles of respiratory syncytial virus subtypes A and B among children hospitalized with bronchiolitis. Pediatr Infect Dis J. 2017;36(8):808‐810. 28383391 10.1097/INF.0000000000001596 PMC5556381

[23] Leung AK , Kellner JD , Davies HD . Respiratory syncytial virus bronchiolitis. J Natl Med Assoc. 2005;97(12):1708‐1713. 16396064 PMC2640754

[24] Riddell CA , Bhat N , Bont LJ , et al. Informing randomized clinical trials of respiratory syncytial virus vaccination during pregnancy to prevent recurrent childhood wheezing: a sample size analysis. Vaccine. 2018;36(52):8100‐8109. 30473186 10.1016/j.vaccine.2018.10.041 PMC6288067

[25] Omer SB , Bednarczyk R , Kazi M , et al. Assessment and validation of syndromic case definitions for respiratory syncytial virus testing in a low resource population. Pediatr Infect Dis J. 2019;38(3):e57‐e59. 30074977 10.1097/INF.0000000000002159 PMC6437080

[26] Ralston S , Hill V . Incidence of apnea in infants hospitalized with respiratory syncytial virus bronchiolitis: a systematic review. J Pediatr. 2009;155(5):728‐733. 19647839 10.1016/j.jpeds.2009.04.063

[27] Jafri HS , et al. Distribution of respiratory syncytial virus subtypes A and B among infants presenting to the emergency department with lower respiratory tract infection or apnea. Pediatr Infect Dis J. 2013;32(4):335‐340. 23337904 10.1097/INF.0b013e318282603a

[28] Sabogal C , Auais A , Napchan G , et al. Effect of respiratory syncytial virus on apnea in weanling rats. Pediatr Res. 2005;57(6):819‐825. 15774838 10.1203/01.PDR.0000157679.67227.11

[29] Khuri‐Bulos N , Lawrence L , Piya B , et al. Severe outcomes associated with respiratory viruses in newborns and infants: a prospective viral surveillance study in Jordan. BMJ Open. 2018;8(5):e021898. 10.1136/bmjopen-2018-021898 PMC5961648 29780032

[30] Moyes J , et al. Epidemiology of respiratory syncytial virus‐associated acute lower respiratory tract infection hospitalizations among HIV‐infected and HIV‐uninfected South African children, 2010–2011. J Infect Dis. 2013;208(Suppl 3):S217‐S226. 24265481 10.1093/infdis/jit479

